# Getting to Grips with Strangles: An Effective Multi-Component Recombinant Vaccine for the Protection of Horses from *Streptococcus equi* Infection

**DOI:** 10.1371/journal.ppat.1000584

**Published:** 2009-09-18

**Authors:** Bengt Guss, Margareta Flock, Lars Frykberg, Andrew S. Waller, Carl Robinson, Ken C. Smith, Jan-Ingmar Flock

**Affiliations:** 1 Department of Microbiology, Swedish University of Agricultural Sciences, Uppsala, Sweden; 2 Department of Microbiology, Tumor and Cellbiology, Karolinska Institutet, Stockholm, Sweden; 3 Department of Bacteriology, Animal Health Trust, Lanwades Park, Kentford, Newmarket, United Kingdom; 4 Department of Pathology and Infectious Diseases, Royal Veterinary College, Hawkshead Lane, North Mymms, Hatfield, Herts, United Kingdom; Children's Hospital Boston, United States of America

## Abstract

*Streptococcus equi* subspecies *equi* (*S. equi*) is a clonal, equine host-adapted pathogen of global importance that causes a suppurative lymphodendopathy of the head and neck, more commonly known as Strangles. The disease is highly prevalent, can be severe and is highly contagious. Antibiotic treatment is usually ineffective. Live attenuated vaccine strains of *S. equi* have shown adverse reactions and they suffer from a short duration of immunity. Thus, a safe and effective vaccine against *S. equi* is highly desirable. The bacterium shows only limited genetic diversity and an effective vaccine could confer broad protection to horses throughout the world. Welsh mountain ponies (n = 7) vaccinated with a combination of seven recombinant *S. equi* proteins were significantly protected from experimental infection by *S. equi*, resembling the spontaneous disease. Vaccinated horses had significantly reduced incidence of lymph node swelling (p = 0.0013) lymph node abscessation (p = 0.00001), fewer days of pyrexia (p = 0.0001), reduced pathology scoring (p = 0.005) and lower bacterial recovery from lymph nodes (p = 0.004) when compared with non-vaccinated horses (n = 7). Six of 7 vaccinated horses were protected whereas all 7 non-vaccinated became infected. The protective antigens consisted of five surface localized proteins and two IgG endopeptidases. A second vaccination trial (n = 7+7), in which the IgG endopeptidases were omitted, demonstrated only partial protection against *S. equi*, highlighting an important role for these vaccine components in establishing a protective immune response. *S. equi* shares >80% sequence identity with *Streptococcus pyogenes*. Several of the components utilized here have counterparts in *S. pyogenes*, suggesting that our findings have broader implications for the prevention of infection with this important human pathogen. This is one of only a few demonstrations of protection from streptococcal infection conferred by a recombinant multi-component subunit vaccine in a natural host.

## Author Summary

Numerous research groups have vaccinated, using recombinant antigens, against streptococcal infections in mouse model systems and shown protection. We have here demonstrated efficient protective vaccination of the natural host, the horse, using recombinant antigens. *Streptococcus equi* subspecies *equi* (*S. equi*) is an equine host-adapted and highly contagious pathogen of global importance. Six out of seven Welsh mountain ponies vaccinated with a combination of seven recombinant *S. equi* proteins were protected from experimental infection as assessed by clinical examination, pyrexia, lymph node swelling, inflammation, bacterial recovery, and post mortem examination. The protective antigens consisted of five surface localized proteins and two endopeptidases that are specific for IgG; the latter were shown to be of major importance for efficacy. Several of the antigens used here have similarities in *Streptococcus pyogenes*, implying that our findings are of importance for development of a vaccine against this important human pathogen.

## Introduction

Access to the genome sequence data of bacterial pathogens permitting the identification of surface exposed and secreted proteins has long been anticipated to revolutionize vaccine design, referred to as reverse vaccinology [1],[2]. However, few vaccines have been taken beyond studies in mouse model systems and shown to confer protection against challenge infection in the natural host.

Strangles, caused by *Streptococcus equi* subsp. *equi* (*S. equi*), is characterized by abscessation of the lymph nodes of the head and neck of the horse and is of significant welfare and economic importance. The development of effective preventative vaccines has been slow. A non-encapsulated strain of *S. equi* (Pinnacle IN™) has been used as a nasal vaccine against strangles, but has not been licensed for sale in Europe due to safety concerns. A second live attenuated vaccine was marketed in Europe [3] (Equilis StrepE), but was withdrawn in 2007. Safety concerns have also been raised over the use of Equilis StrepE [4],[5]. A safe and effective vaccine against *S. equi* is thus highly desired.

*S. equi* evolved from an ancestral strain of *S. equi* subsp. *zooepidemicus* (*S. zooepidemicus*). The population of the *S. zooepidemicus* group is extremely diverse and consists of at least 218 sequence types, whereas isolates of *S. equi* from the USA, Canada, Australia and Europe are either ST-179 or a single locus variant, ST-151 [6] (http://pubmlst.org/szooepidemicus/). The limited genetic diversity of *S. equi* suggests that an effective vaccine could confer broad protection to horses throughout the world.

We have demonstrated previously that vaccination of Welsh mountain ponies with EAG [7],[8], SclC [9] and CNE [10] (Trivacc) conferred partial protection against challenge by *S. equi* [11]. The amount of nasal discharge, the number of bacteria recovered from nasal washes and the occurrence of abscess material (empyema) in guttural pouches, following rupture of abscesses formed in the retropharyngeal lymph nodes, differed significantly between the vaccinated group and a non-vaccinated control group. However, clinical scoring and mean rectal temperatures did not differ significantly. This experiment thus showed that parameters of importance for spreading disease between horses were reduced significantly, but that the level of protection in individual horses was limited [11]. Using a set of seven recombinant proteins (Septavacc), we demonstrate here that 85% protection was obtained.

## Results

### Choice of antigens and antibody response

Five of the antigens in the Septavacc composition are predicted to be localized on the surface of *S. equi* (EAG [7],[8], CNE [10], SclC [12], SEQ0256 and SEQ0402 [13]) through sortase-mediated attachment to the peptidoglycan cell wall. EAG binds to albumin, α-2 macroglobulin (A2M) and IgG [8],[14]. CNE binds to collagen [10], and is located within the FimI pilus locus of *S. equi* and *S. zooepidemicus* [13],[15]. SclC is a member of a collagen-like protein family, which in *S. equi* consists of seven members, each with a unique N-terminal domain of unknown function [12]. The proteins encoded by SEQ0256 and SEQ0402 contain features typical of cell surface anchored proteins and an N-terminal non-repetitive domain. The N-terminal domains were used in this study, the functions of which are unknown and neither shows homology to any characterized protein. The two additional antigens in Septavacc, IdeE and IdeE2 are IgG endopeptidases, where IdeE2 has greater activity towards horse IgG. Both IdeE and IdeE2 are predicted to be secreted [16],[17] and IdeE has an antiphagocytic activity by binding directly to neutrophils [17].

The antigens in Septavacc were selected from a larger antigen pool based on the level of protection conferred in an experimental mouse model of strangles. Mice were immunized with recombinant antigens either individually, or in combination, and subsequently experimentally infected with *S. equi*. Following challenge, mice were examined daily for loss of weight and for nasal colonization in comparison with non-vaccinated controls. The reproducibility of the model is sufficiently robust to allow comparison between different experiments. A ranking list based on the protective efficacy of the different antigens could therefore be generated (Table 1). Vaccination with FNE, SFS and FNEB did not result in protection although good antibody titers were obtained with these antigens [18]. Good immune responses were obtained to all other antigens with the exception of IdeE2 and all of these antigens conferred significant protection in mice (Table 1).

**Table 1:** Antigens used to vaccinate mice.

Exp. number	Antigen	Relative protective efficacy in mouse model	Reference to antigen description or use as vaccine
1	FNE	−	[37]
2	SFS	−	[38]
3	FNEB	−	[18],[20]
4	EAG	+	[7],[20]
5	CNE	+	[10],[20]
6	SEQ0936, (putative pili)	+	This study
7	SEQ0944, (pullulanase)	+	This study
8	SEQ0256	+	This study
9	IdeE	+	[16]
10	CNE+EAG	++	[20]
11	SclC	++	[12],[20]
12	EAG+SFS+FNE	++	[20]
13	EAG+CNE+SclC	++	[11]
14	IdeE2	+++	[19]
15	IdeE+IdeE2+EAG	+++	This study
16	SEQ0402	+++	This study
17	SEQ0256+SEQ0402	+++	This study

Ranking of efficacy on a range from − to +++ was based on weight loss and nasal colonization of mice challenged with *S. equi* after vaccination with indicated antigens.

**Figure 1:**
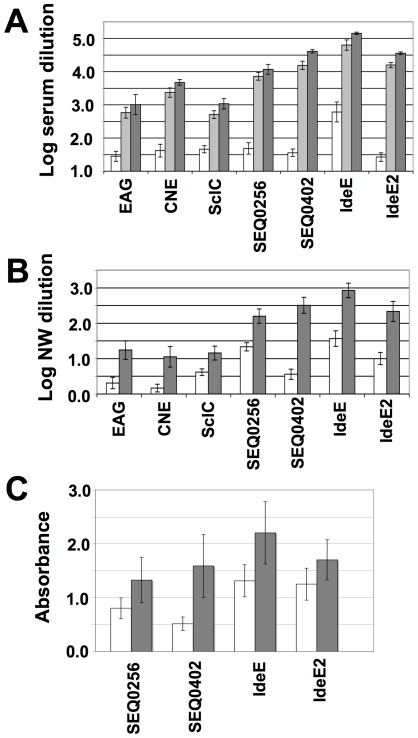
Determination of antibody titers in ponies vaccinated with Septavacc. Serum samples (A) and nasal washes (B) were taken from vaccinated ponies before immunization (white bars) or 12 days after third immunization (dark grey bars). Serum samples were also taken 11 days after second vaccination (light grey bars in panel a). Microtiter plates were coated with indicated antigens and ELISA was performed as described earlier [7]. The dilutions required to obtain an absorbance of 1.0 for nasal wash samples and 1.5 for sera were calculated; the titration curves are linear and parallel at these values. The average and standard error of the mean (SEM) of log values of these dilutions are plotted. All increases of IgG titers are significant. IgA was determined using nasal washes diluted 2-fold and the absorbance value plotted; white bars before immunization and grey bars after third vaccination (C).

Seven Welsh mountain ponies were vaccinated with Septavacc and an additional seven ponies were given adjuvant only as control via both the subcutaneous and intranasal routes, followed by experimental infection with 1×10^8^ colony forming units (cfu) of *S. equi* strain 4047. Antibody responses against the antigens in serum samples and nasal washes were analyzed by ELISA (Figures 1A, B and C). All ponies responded well and it was noted that IgG responses in nasal washes and in sera had low correlation in individual ponies (R^2^ from 0.01 to 0.28); a pony could respond well in sera but less so in nasal washing and v.v. This implies the generation of independent immune responses in mucosa and sera, possibly as a result of the two routes of immunization employed. Exudation of IgG from sera to mucosal surfaces presumably also contributes to mucosal IgG. A high background level of IgG (in pre immune sera) against IdeE is presumably due to non-immunologic binding to IdeE. IgA responses in nasal washes against SEQ0256, SEQ0402, IdeE and IdeE2 were moderate but significant for SEQ0402, IdeE and IdeE2 with p values 0.04, 0.02 and 0.05 respectively.

**Figure 2:**
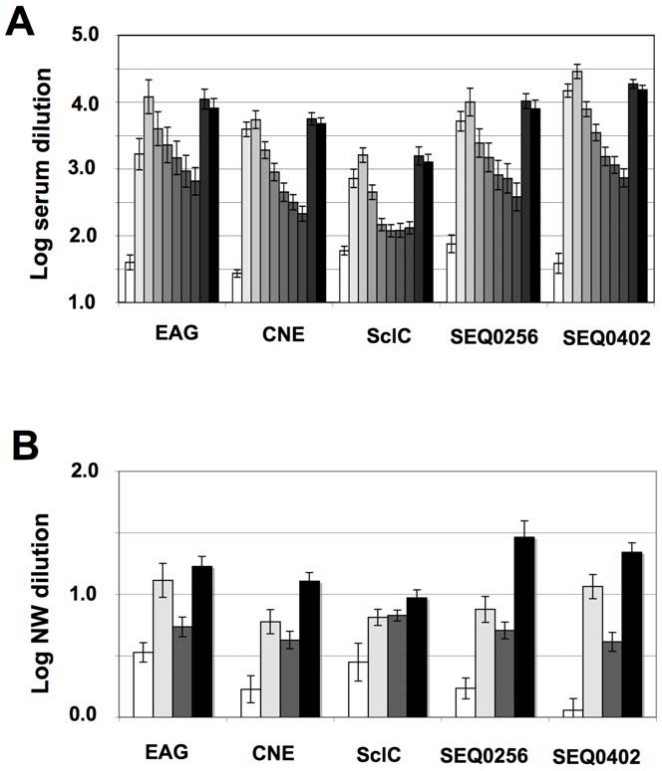
Determination of antibody titers in ponies vaccinated with Pentavacc. ELISA was performed and values calculated as in Figure 1 showing average and SEM. Serum samples (A) were taken on days 1, 71, 86, 127, 155, 183, 211, 239, 281, and 288 shown by gradually darker bars. Immunizations were given on days 4, 60, 74, and 270 and challenge on day 284. The white bar is thus from pre-immunization sample and the last black bar from 4 days after challenge. Comparison of titers from pre-immunization (day 1) and the last before V4 (day 239) gives p-values of 0.0002, 0.0001, 0.010, 0.014, and 0.0001 for the five antigens. Nasal wash samples (B) were taken on days 1, 127, 239 and 281 (white, light grey, dark grey and black bars respectively). Comparison of titers from pre-immunization (day 1) and before V4 (day 239) gives p-values of 0.08, 0.01, 0.036, 0.001, and 0.0006 for the five antigens.

In a separate study, seven ponies were immunized with a Pentavacc formulation, containing the same five surface antigens as Septavacc, but with the omission of IdeE and IdeE2. Serum samples and nasal washes were taken every month. Significantly elevated IgG levels to the five surface proteins could be detected 6 months post V3 and an additional booster (V4) on day 270 led to a rapid increase of IgG in sera against all antigens (Figure 2A). IgG against the antigens in nasal washes also persisted for a long time as shown in Figure 2B.

**Figure 3:**
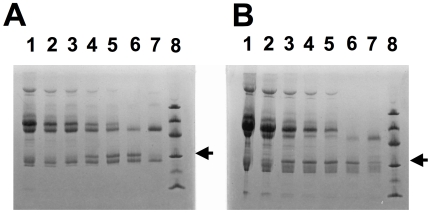
SDS-PAGE of IgG cleavage with endopeptidase IdeE and IdeE2. (A) IdeE cleavage of human IgG in the presence of serum from Septavacc vaccinated ponies and (B) IdeE2 cleavage of horse IgG in the presence of serum from Septavacc vaccinated ponies. (A) Lane 1; Septavacc diluted 4 times, lane 2; Septavacc serum diluted 8 times, lane 3; Septavacc serum diluted 16 times, lane 4; Septavacc serum diluted 32 times, lane 5; Septavacc serum diluted 64 times, lane 6; human IgG+IdeE, lane 7; human IgG, lane 8; size marker (in kDa 97, 66, 45, 30, 20, 14,4). The same amount of IdeE is added to sample 1–6 and the same amount of human IgG is added to sample 1–7. (B) Lane 1; Septavacc undiluted, lane 2; Septavacc diluted 2 times, lane 3; Septavacc diluted 4 times, lane 4; Septavacc diluted 8 times, lane 5; Septavacc diluted 16 times, lane 6; horse IgG+IdeE2, lane 7; horse IgG, lane 8; size marker. The same amount of IdeE2 was added to sample 1–6 and the same amount of horse IgG was added to sample 1–7. The arrows just above the 30 kDa band indicate the cleavage product of IgG.

Sera from Septavacc vaccinated ponies were also tested for the ability to inhibit the IgG cleaving activity of IdeE and IdeE2. Sera pooled from ponies immunised with Septavacc could be diluted 16 fold and still inhibit the cleavage of human IgG by IdeE. The inhibitory activity against IdeE2 cleavage was much less. At a 2 fold serum dilution the cleavage of horse IgG was inhibited (Figure 3A and B). Neither pre-immune sera from the Septavacc vaccinated ponies nor sera from Pentavacc vaccinated ponies had any effect on the IgG cleavage activity of IdeE or IdeE2 (data not shown).

### Clinical assessment of infection

**Figure 4:**
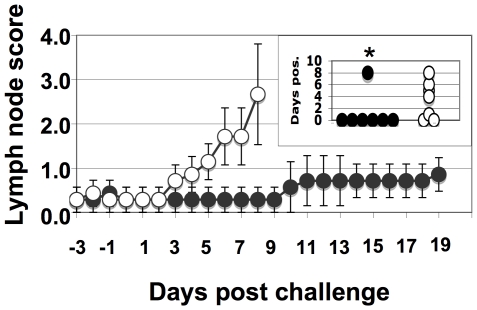
Lymph node swelling over time in Septavacc and control ponies. Lymph node swelling was monitored from three days pre-challenge (day 85) using an arbitrary scale from 0 to 4. Average values and SEM are shown. The insert shows number of days each pony was considered positive, i.e. with a value exceeding 2. P-value in insert: *p = 0.05. Non-vaccinated (open symbols) (n = 7) and Septavacc vaccinated (closed symbols) (n = 7).

The swelling and abscessation of submandibular lymph nodes is a typical clinical sign of infection with *S. equi*. The mean lymph node scores in Septavacc vaccinated ponies differed from the control group and the number of days where an individual pony's score exceeded 2, differed significantly (p = 0.0013) (Figure 4).

**Figure 5:**
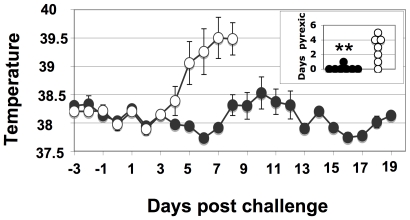
Temperature over time in Septavacc and control ponies. Mean rectal temperature was monitored from three days pre-challenge (day 85). Average values and SEM are shown. The insert shows number of days each pony was considered pyrexic, i.e. with a temperature exceeding 39.0°C. P-value in insert: **p = 0.01. Non-vaccinated (open symbols) (n = 7) and Septavacc vaccinated (closed symbols) (n = 7).

The normal rectal temperature of Welsh mountain ponies is 37–38°C and a pony with a rectal temperature of 39°C or higher is considered pyrexic. All ponies in the control group became pyrexic at some stage during challenge compared to only one pony in the Septavacc vaccinated group. The accumulated number of days that individual ponies in the vaccinated or control groups were pyrexic was 5 and 30 days, respectively (p = 0.0001) (Figure 5).

**Figure 6:**
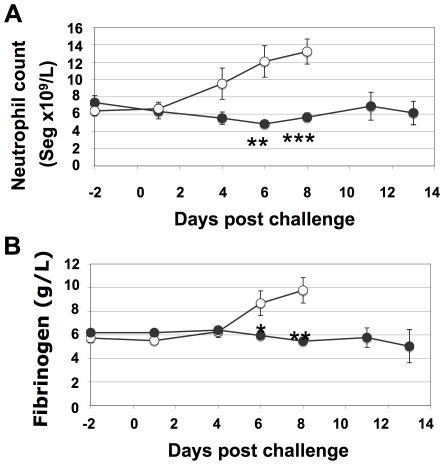
Inflammatory markers over time in Septavacc and control ponies. Neutrophil counts (A) and fibrinogen levels (B) were monitored from two days pre-challenge (day 86). Average values and SEM are shown. P-values: **p = 0.002, ***p = 0.0004 in (a), *p = 0.024, **p = 0.002 in (B). Non-vaccinated (open symbols) (n = 7) and Septavacc vaccinated (closed symbols) (n = 7).

Infection with *S. equi* leads to a systemic inflammation, manifested as an increase in blood fibrinogen and neutrophil levels. As shown in Figure 6A and B, fibrinogen and neutrophil levels of ponies vaccinated with Septavacc remained normal, whereas the non-vaccinated group had significantly higher mean values.

Ponies vaccinated with Pentavacc, containing the same antigens as in Septavacc but omitting the IgG endopeptidases, were challenged 14 days after the last booster. These ponies differed from the corresponding control group in terms of elevated temperature, fibrinogen levels and nasal discharge, but not significantly so. One pony was protected fully.

### Post mortem scoring

To minimize suffering and in accordance with our strict ethical and welfare code, ponies were euthanized as soon as clinical signs of *S. equi* infection became apparent. All of the control ponies were euthanized between 8 to 12 days post challenge. Vaccinated ponies, however, had reduced clinical signs and all ponies reached the end of the study, 21 days post challenge. Following euthanasia, all of the ponies were subject to post mortem examination to quantify the level of pathology observed using a scoring system as described in Materials and Methods.

**Figure 7:**
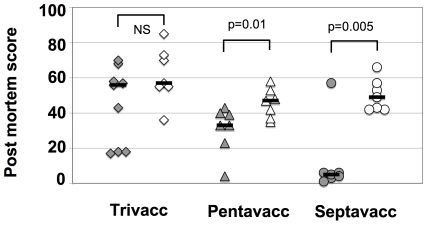
Post mortem score. Ponies were vaccinated with three different antigen combinations, (Trivacc, Pentavacc and Septavacc) followed by challenge with *S. equi*. Post mortem scoring was performed using a scoring system described in Materials and Methods. Non-vaccinated (open symbols) and vaccinated (closed symbols) for individual ponies and median values are shown. Mann Whitney test was used to calculate significance.

**Figure 8:**
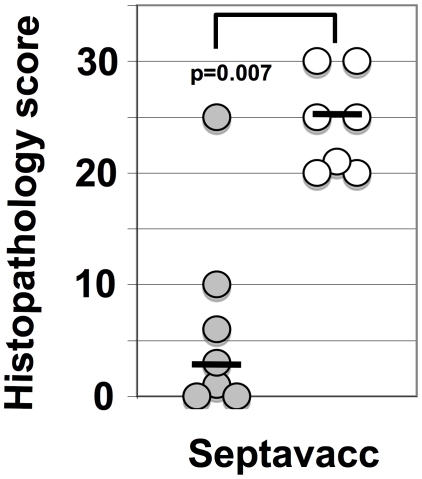
Histopathological examinations of Septavacc and control ponies. Evaluation was done using the scoring system described in Materials and Methods. Non-vaccinated (open symbols) and vaccinated (closed symbols) for individual ponies and median values are shown. Mann Whitney test was used to calculate significance.

Figure 7 summarizes the individual post mortem scores of ponies vaccinated with Trivacc (EAG, SclC, CNE) [11], Pentavacc and Septavacc, containing three, five and seven antigens respectively. Increasing the number of antigens comprising each vaccine significantly reduced the post mortem score. Only one of the ponies vaccinated with Septavacc had lymph node abscesses, compared with abscesses in all seven non-vaccinated ponies. To confirm these gross pathological findings, samples from ponies vaccinated with Septavacc were examined histopathologically and scored using a system as described in Materials and Methods. Again, significant differences were seen between the Septavacc and control groups (p = 0.006) (Figure 8). Histopathological examination of the left and right retropharyngeal and submandibular lymph nodes identified 19 lymph node abscesses in the control ponies and 3 lymph node abscesses in a single pony in the Septavacc group (p = 0.00001). Seventy-eight and 10% of all lymph nodes were culture positive for *S. equi* in the control and vaccine groups respectively (p = 0.004).

## Discussion

Taking all of the results together, vaccination with Septavacc resulted in 85% protection from disease, with only one vaccinated pony out of seven developing lymph node abscesses.

This study is one of only a few demonstrations of protection in a natural host from streptococcal infection conferred by a recombinant multi-component subunit vaccine. No significant adverse effects were seen in any of the vaccinated ponies, demonstrating that both the recombinant antigens and the adjuvant were safe. It is also clear that the antiphagocytic capsule and other immune evasion mechanisms employed by *S. equi* were not sufficient to counter successful vaccination with the recombinant proteins used here. The approach taken here is significantly safer than currently available live attenuated strains of *S. equi*.

The large difference in efficacy between Pentavacc and Septavacc (p = 0.036 for post mortem scoring), suggests that the inclusion of one or both of the endopeptidases IdeE and IdeE2 is important for protection in the natural host. As antibodies in sera from the Septavacc vaccinated ponies can inhibit the IgG cleaving activities of IdeE and IdeE2, it is conceivable that these antibodies prevent the destruction of antibodies directed against the other five components in Septavacc .We have recently found that immunization of mice with IdeE and IdeE2 as single antigens confer protection in the mouse model of strangles [19]. We assume this effect might be due to opsonising antibodies targeting IdeE and IdeE2 during the secretion process. It cannot be ruled out that the difference in efficacy between Pentavacc and Septavacc might also be attributed to the different vaccination schedules. The ponies vaccinated with Pentavacc were infected 6.5 months later than those vaccinated with Septavacc but were therefore given an additional booster (V4) two weeks before challenge. After this booster, antibody titers in these ponies reached the same level as in ponies vaccinated with Septavacc. With the exception of IdeE and IdeE2, the contribution of each protein to the protective activity of Septavacc has not been addressed in this study. Neither have we attempted to analyze in depth the mechanism of action of the protective antibodies. The activity of antibodies against surface localized antigens might de dual: adherence blocking and opsonic. We have previously found that antibodies against CNE effectively block adherence of *S. equi* to collagen [20].

With the exception of the 5′ variable region of SeM [21] the genomic variation between isolates of *S. equi* is thought to be minimal and all strains analyzed to date by MLST are either ST-179 or the single locus variant ST-151. The antigens used in this study were all cloned from the Swedish *S. equi* strain 1866, which is SeM type-9, whilst the *S. equi* strain 4047 challenge strain is SeM type-3 and was isolated in the UK. Therefore, it is likely that the antigens present in Septavacc will confer broad protection against *S. equi* strains from around the world.

*S. equi* shares >80% sequence identity with *Streptococcus pyogenes* [13] and several components utilized in our studies share similarity with *S. pyogenes* antigens, either by homology or function. The *S. pyogenes* gene encoding the collagen binding protein Cpa is located in the variable FCT region (fibronectin- and collagen-binding T-antigen) and is part of a pilus-like structure [22]. Similarly, *cne* is located in a pilus locus (FimI) that includes genes encoding SrtC.1 and a putative backbone pilus subunit suggesting that CNE is also attached to a pilus-like structure [13]. EAG, like GRAB from *S. pyogenes*, binds the proteinase inhibitor A2M [8],[23],[24]. SclC is one of seven collagen-like surface proteins in *S. equi*, whilst *S. pyogenes* genomes contain two such putative proteins, SclA and SclB [9]. The IgG-specific endopeptidases used here, IdeE and IdeE2 [19], are similar both in function and amino acid sequence to IdeS/Mac/sib35 of *S. pyogenes* [16],[17],[25],[26]. Antibodies against IdeS in convalescent patients were able to neutralize its function [27]. Interestingly, Cpa (plus other pili components) and Sib35 have been identified as protective antigens in mouse models of *S. pyogenes* infection. The Cpa combination and Sib35 gave good protection in a mouse system whereas antibodies against GRAB could only opsonize capsule-deficient mutants of *S. pyogenes* [28]–[30]. Thus, it is conceivable that vaccination of humans with a combination of *S. pyogenes* antigens similar to the ones used in Septavacc could prove effective against this important human pathogen

In a study by Timoney *et al.*, [31] two combinations of recombinant proteins derived from *S. equi* (SzPSe, CNE, Se51.9, Se44.2 and Se46.8 or SeM, Se44.2, Se75.3, Se42.0, Se110.0 and Se18.9) were tested as vaccines against strangles. However, neither combination protected horses from infection with *S. equi* [31]. Two of these proteins CNE and IdeE2 (Se44.2) are included in the Septavacc vaccine, suggesting that the additional components of Septavacc are important in generating a protective immune response. It was also suggested that an effective strangles vaccine should result in immune-mediated tonsillar clearance since tonsillar adherence is a crucial early step in the pathogenesis of strangles [31],[32]. If this is the case, the route of immunization and choice of adjuvant, which differ between these studies, might be of utmost importance. We have generally noted that mice immunized by the intranasal route are far better protected than those immunized subcutaneously. IgA responses in nasal washes were obtained in this study. It is likely that the ability to block bacterial adherence to mucosal surfaces is of importance to the protection observed in this study. A good mucosal immune response was obtained in the mouse by using nanospheres to which *S. equi* proteins, extracted from the bacterial wall, were adsorbed [33],[34]. The poorer efficacy observed with Trivacc could be due, not only to fewer antigens, but also to the fact that immunizations were only intra muscular and i.n. In this study, immunizations were both subcutaneous, near the retropharyngeal lymph node, and i.n. The necessity of both routes of immunizations will be determined in future studies.

In conclusion, we have demonstrated that using recombinant antigens, a protective immune response against a streptococcal infection can be obtained in the natural host, not just in a mouse model system. Immune protection by vaccination does not necessarily require an attenuated live vaccine or vaccination with killed bacteria, conventional strategies for vaccine design.

## Materials and Methods

### Ethics statement

Approval for mice experiments was obtained from Swedish Animal Welfare Agency. The pony studies were conducted under a Home Office Project License. The Animal Health Trust Ethical Review Committee approved the Research Program Proposal for these studies.

### Bacterial strains, plasmids and growth conditions

*S. equi* strain 1866 SeM type-9 (obtained from Nordvacc Läkemedel AB, Sweden) was used as source DNA for cloning the antigens and used as the challenge strain in mouse infection experiments. The *S. equi* strain 4047, SeM type-3 was isolated from a case of strangles (Animal Health Trust), was used as the challenge strain in horse infection experiments. To clone and express the recombinant antigens, SEQ0256, SEQ0402, SEQ0944, SEQ0936, and IdeE2, the *E. coli* strains ER2566 or BL21(DE3) and plasmid vector pTYB4, belonging to the IMPACT™ Protein Purification System (New England Biolabs Inc., MA) were used. The *E. coli* strain BL21(DE3) and plasmid vector pGEX-6P-1 belonging to GST-glutathione affinity system (GE Healthcare) were used to clone and express IdeE. Plasmids and PCR amplified DNA fragments were purified using the QIAprep Spin Miniprep (Qiagen, Hilden, Germany).

Strains of *S. equi* were grown on blood agar plates or in Todd-Hewitt broth (Oxoid, Basingstoke, Hampshire, United Kingdom) and *E. coli* clones were cultured in Luria-Bertani (LB) broth, supplemented with ampicillin (50 µg/ml), or on LAA plates [LB-broth with ampicillin and agar (15 g/L)]. Incubations were at 37°C unless otherwise stated.

### Construction of clones

The *S. equi* strain 4047 genome database (www.sanger.ac.uk/) was analyzed to select open reading frames encoding a number of extra cellular proteins to be tested as potential antigens in the vaccination trials. To identify the predicted signal sequences, the computer program SignalP (http://www.cbs.dtu.dk/services/SignalP/) was used.

Chromosomal DNA from *S. equi* strain 1866 was used as a template to PCR amplify the genes (or part of the genes) encoding SEQ0256, SEQ0402, SEQ0944, SEQ0936, IdeE, and IdeE2. The sequences of primers used are listed in Table 2. Cleavage sites for restriction enzymes were included in the primer sequences to match the cloning sites in the plasmid vectors. The PCR amplifications were performed using the primers (20 pmol/µl) and ReadyToGo™ PCR beads (GE Healthcare) using a standard PCR programme (Step 1, pre-heat 1 minute at 95°C, DNA strand separation; Step 2, 30 seconds at 95°C; Step 3, annealing 15 seconds at usually 5 degree below the melting temperature; and Step 4, elongation for 2 minutes at 72°C, Steps 2–4 were run for 30 cycles.) The PCR products were analyzed on an agarose gel, and thereafter purified using the QIAquick PCR Purification Kit™ (Qiagen). After restriction enzyme cleavage the fragments were purified again using the kit mentioned above before ligation into the respective vector using the ReadyToGo T4DNA Ligase (GE Healthcare). After ligation, the samples were transformed into competent *E. coli* strain ER2566 or BL21(DE3) using electroporation, spread on LAA plates and incubated over night at 37°C. Next day, a number of colonies were analyzed by PCR for the presence of inserts, using the respective primer combination. Clones with correct inserts were further analyzed by DNA sequencing.

**Table 2:** The primer sequences used to PCR amplify the genes *seq0936, seq0944, seq0256, seq0402*, *ideE2* and *IdeE2*.

Gene	Corresponding part in protein	Primer name	Sequence
*seq0936*	1–422	Eqp541	GCAT**CCATGG**ATACAGCAAGCTATACCA
		Eqp542	TCGA**CTCGAG**GATAGACCCTTTTTTATTAAC
*seq0944*	1–427	Eqp271	GCAG**CCATGG**AGAGTCTGACGAGTGTTGA
		Eqp272	TCAC**CTCGAG**TCCTAGCTCACCGTCATAAGC
*seq0256*	1–441	Eqp51	GTAG**CCATGG**AAACGACTACTGCTAGTGCA
		Eqp52	CTGG**CTCGAG**CGGTTTAGCAACCAAGGCT
*seq0402*	1–196	Eqp81	CATG**CCATGG**CGACTACCCTAGCAGGACAAA
		Eqp82	CTAG**CTCGAG**GTGCTTAAGCTTTTCAATCTG
*IdeE*	1–315	IdeG1	TACT**GGATCC**GACGATTACCAAAGGAATGCTAC
		IdeG2	TGAT**CTCGAG**TTAGCTCAGTTTCTGCCATATG
*IdeE2*	1–344	Ide2F	CATG**CCATGG**AGGTAGTTGAAGTTTGGCCTAAT
		Ide2R	CCG**CTCGAG**TTTTTCTGTGTTGTTGAAGTAATCTGC

The bold nucleotides correspond to the introduced restriction cleavage sites. The number indicated in the corresponding protein refers to amino acids in the mature protein.

### Production of recombinant antigens

To produce and purify the recombinant proteins SEQ0256, SEQ0402, SEQ0936, SEQ0944, [13] and IdeE2, the expression and purification system IMPACT™ T7 (NEB) was used. Briefly, following the manufacturer's instructions the clones containing the recombinant plasmids were grown at 37°C in LB media supplemented with ampicillin (final conc. 50 µg/ml). At an optical density (OD_600 nm_)∼0.6, the growth media were supplemented with IPTG (final conc. 0.3 mM) and the growth temperature shifted to 20°C. After incubation over night the cells were harvested and resuspended in a buffer [20 mM Tris-HCl (pH 8.0), 500 mM NaCl, 0.1 mM EDTA, and 0.05% (v/v) TWEEN20] and lysed by freezing and thawing. After centrifugation, the supernatants were sterile filtrated and applied onto a chitin column. The columns were washed extensively using the same buffer and treated subsequently with cleavage buffer [20 mM Tris-HCl (pH 8.0), 50 mM NaCl, 0.1 mM EDTA, and 30 mM dithiothreitol (DTT)]. The eluted samples containing the antigens were dialysed against phosphate-buffered saline [PBS; 137 mM NaCl, 2.7 mM KCl, 10 mM Na_2_HPO_4_, 1.4 mM KH_2_PO_4_ (pH 7.4)].

Recombinant IdeE was produced using the GST-glutathione affinity system. Briefly, according to the procedure described above, after growth, induction and harvest, the *E. coli* cells were suspended in PBS supplemented with TWEEN20, final conc. 0.1% (v/v) (PBST) whereupon the cells were lysed by freezing and thawing. After centrifugation, the supernatant was sterile filtrated and batch purified with Glutathione-sepharose beads. After extensive washing using PBST the fusion protein was treated with scissor protease to release IdeE. Finally, the amounts of antigens obtained were determined using spectrophotometry and the quality analyzed by SDS-PAGE coomassie staining. The proteins were stored finally at −20°C. IdeE and IdeE2 were produced as full-length protein with a few extra amino acids from the vectors and both proteins showed IgG-cleaving activity. It should be noted that IdeE2 had a tendency to partially precipitate upon freezing.

The production of EAG, SclC and CNE has been described previously [11].

### IgG cleaving activity by IdeE and IdeE2

The cleaving activity of IdeE was assayed against human IgG (Bethyl Laboratories inc., Montgomery, TX) whereas the IdeE2 activity was measured against horse IgG (Bethyl Laboratories inc., Montgomery, TX). Before cleavage, DTT was added to the endopeptidases to a final concentration of 0.5 mM. Thereafter the enzymes were added to IgG (1 mg/ml) for 30–60 minutes and cleavage was visualised by coomassie blue staining of SDS-PAGE gels.

To test for inhibitory activity of sera against the endopeptidases the same type of assay as above was used. Briefly, Septavacc sera from six ponies were pooled and diluted in steps of two. As controls, pre-immune Septavacc sera and Pentavacc sera were also pooled and diluted. The assay was performed by mixing 2 µl of either endopeptidase (15 µg/ml) separately with 2 µl of diluted sera for 15 min in room temperature after which 8 µl of IgG (1 mg/ml) was added and incubated for 45 minutes at 37°C and the samples analysed on SDS-PAGE.

### Mouse model of strangles

Prior to experimental challenge infection, female NMRI mice (n = 15) were vaccinated with recombinant proteins derived from *S. equi* as described earlier [20]: 12 µg of each antigen and 10 µg of adjuvant (Abisco 300, Isconova, Uppsala, Sweden [35]) were given intranasally on days 1, 14 and 21. Control mice (n = 15) were given adjuvant only. Blood samples were collected for assessment of antibody titers in ELISA [20].

Infection of mice with *S. equi* strain 1866 was performed 7 days after the final booster as described previously [20]. Briefly, 10^6^ CFU of *S. equi* strain 1866 (cultivated in THB+10% horse serum+1% yeast extract for 4 hours in a 5% CO_2_ enriched atmosphere) were given intranasally to anesthetized mice. Weight loss and colonization of nostrils were followed daily. Blood agar plates containing gentiana violet, to select for streptococcal growth, were placed gently onto the nostrils. Bacteria were spread out on the plates, which were then incubated overnight in a 5% CO_2_ enriched atmosphere. A scoring system was used where 0–5 colonies = 0, 5–100 = 1, >100 colonies = 2 and confluent growth = 3.

### Immunization of ponies

Healthy Welsh Mountain Ponies (n = 7) were vaccinated with Septavacc via administration of 1 ml subcutaneous (s.c.) injections bilaterally close to the retropharyngeal lymph nodes and 2 ml intranasally (i.n.) by spraying into each nostril on days 4, 60, and 74 (V1, V2 and V3). The Septavacc vaccine doses contained 150 µg for i.n. and 50 µg for s.c. injections of each antigen (EAG, CNE, SclC, SEQ0256, SEQ0402, IdeE, and IdeE2). Abisco 300 (Isconova, Uppsala, Sweden [35]) (500 µg per i.n. dose) and Abisco 200 (375 µg per s.c. dose) were used as adjuvants. Septavacc ponies were challenged on day 88. Pentavacc vaccinated ponies (n = 7) followed the same vaccination protocol as above, but were given an additional booster vaccination (V4) on day 270 and challenged on day 284. Negative control ponies were given adjuvant only, mixed with PBS (n = 7). Sera and nasal washes were taken regularly to quantify IgG responses by ELISA [11]. IgA was determined in nasal washes at a 2-fold dilution, using mouse anti horse-IgA monoclonal antibody (Serotec, Oxford, UK) followed by rabbit anti mouse-IgG HRP conjugated antibodies for detection (Dako, Denmark).

### Experimental infection of ponies

Ponies were transferred to a containment unit three days before challenge. Two weeks after the final booster immunization, each pony was challenged with *S. equi* strain 4047 administered via the spraying of a 2 ml culture containing 5×10^7^ cfu into each nostril. Bacteria were grown overnight in Todd Hewitt broth and 10% fetal calf serum (THBS) in a 5% carbon dioxide enriched atmosphere at 37°C, diluted 40-fold in fresh pre-warmed THBS, further cultivated and harvested at an OD = 0.3. This infection dose has been shown to optimize the infection rate, whilst avoiding overwhelming the host immune response, as determined in previous studies [11],[36].

### Clinical evaluation of and sampling from ponies

Ponies were monitored for the onset of clinical signs of disease over a period of three weeks post challenge by daily physical examination, rectal temperature, lymph node swelling and nasal discharge scoring. Blood samples were taken for evaluation of fibrinogen concentration as described in [11] and neutrophil levels by total white blood count performed on Beckman-Coulter ACTdiff analyzer with a manual differential count to calculate % neutrophils.

The level of swelling of SMLNs was defined as 0 = normal, 1 = slight swelling, 2 = moderate swelling, 3 = severe swelling and 4 = abscessated [11]. Bilateral swelling of submandibular lymph nodes was scored separately

### Post mortem examination

Post mortem examination was performed on all ponies following the onset of clinical signs of infection or on reaching the study endpoint at 3 weeks post challenge. The severity of disease pathology was quantified according to a scoring system described previously [11]. The severity of disease on histopathological examination was scored according to the following scoring system: empyema of guttural pouch 5, lymph node abscessation 5, pharyngitis 1, lymphadenitis 1, and rhinitis 1.

### Statistics

Fischer's exact test was used for comparison of values from arbitrary scoring using a cut-off value splitting the group into “low/negative” or “high/positive”. Cut-off values were for nasal colonization in mice 1.5; for lymph node scoring 2; for pyrexia 39°C. Mann Whitney test was used for post mortem and histopathology scoring in ponies. T-test was used to compare temperatures, fibrinogen and neutrophil levels in ponies and weight loss in mice.

## References

[1] Bambini S, Rappuoli R (2009). The use of genomics in microbial vaccine development.. Drug Discov Today.

[2] Serruto D, Serino L, Masignani V, Pizza M (2009). Genome-based approaches to develop vaccines against bacterial pathogens.. Vaccine.

[3] Jacobs AA, Goovaerts D, Nuijten PJ, Theelen RP, Hartford OM (2000). Investigations towards an efficacious and safe strangles vaccine: submucosal vaccination with a live attenuated Streptococcus equi.. Vet Rec.

[4] Kemp-Symonds J, Kemble T, Waller A (2007). Modified live Streptococcus equi (‘strangles’) vaccination followed by clinically adverse reactions associated with bacterial replication.. Equine Vet J.

[5] Newton R, Waller A, King A (2005). Investigation of suspected adverse reactions following strangles vaccination in horses.. Vet Rec.

[6] Webb K, Jolley KA, Mitchell Z, Robinson C, Newton JR (2008). Development of an unambiguous and discriminatory multilocus sequence typing scheme for the Streptococcus zooepidemicus group.. Microbiology.

[7] Flock M, Jacobsson K, Frykberg L, Hirst TR, Franklin A (2004). Recombinant Streptococcus equi proteins protect mice in challenge experiments and induce immune response in horses.. Infect Immun.

[8] Lindmark H, Jonsson P, Engvall E, Guss B (1999). Pulsed-field gel electrophoresis and distribution of the genes zag and fnz in isolates of Streptococcus equi.. Res Vet Sci.

[9] Karlstrom A, Jacobsson K, Guss B (2006). SclC is a member of a novel family of collagen-like proteins in Streptococcus equi subspecies equi that are recognised by antibodies against SclC.. Vet Microbiol.

[10] Lannergård J, Frykberg L, Guss B (2003). CNE, a collagen-binding protein of Streptococcus equi.. FEMS Microbiol Lett.

[11] Waller A, Flock M, Smith K, Robinson C, Mitchell Z (2007). Vaccination of horses against strangles using recombinant antigens from Streptococcus equi.. Vaccine.

[12] Karlström Å, Jacobsson K, Flock M, Flock J-I (2004). Identification of a novel collagen-like protein, SclC, in Streptococcus equi using signal sequence phage display.. Vet Microbiol.

[13] Holden MT, Heather Z, Paillot R, Steward KF, Webb K (2009). Genomic evidence for the evolution of Streptococcus equi: host restriction, increased virulence, and genetic exchange with human pathogens.. PLoS Pathog.

[14] Jacobsson K, Jonsson H, Lindmark H, Guss B, Lindberg M (1997). Shot-gun phage display mapping of two streptococcal cell-surface proteins.. Microbiol Res.

[15] Beres SB, Sesso R, Pinto SW, Hoe NP, Porcella SF (2008). Genome sequence of a Lancefield group C Streptococcus zooepidemicus strain causing epidemic nephritis: new information about an old disease.. PLoS ONE.

[16] Lannergard J, Guss B (2006). IdeE, an IgG-endopeptidase of Streptococcus equi ssp. equi.. FEMS Microbiol Lett.

[17] Timoney JF, Yang J, Liu J, Merant C (2008). IdeE reduces the bactericidal activity of equine neutrophils for Streptococcus equi.. Vet Immunol Immunopathol.

[18] Lannergard J, Flock M, Johansson S, Flock JI, Guss B (2005). Studies of fibronectin-binding proteins of Streptococcus equi.. Infect Immun.

[19] Hulting G, Flock M, Frykberg L, Lannergård J, Flock J-I (2009). Two novel IgG endopeptidases of Streptococcus equi FEMS Microbiol Letters: In press..

[20] Flock M, Karlstrom A, Lannergard J, Guss B, Flock JI (2006). Protective effect of vaccination with recombinant proteins from Streptococcus equi subspecies equi in a strangles model in the mouse.. Vaccine.

[21] Kelly C, Bugg M, Robinson C, Mitchell Z, Davis-Poynter N (2006). Sequence variation of the SeM gene of Streptococcus equi allows discrimination of the source of strangles outbreaks.. J Clin Microbiol.

[22] Nakata M, Koller T, Moritz K, Ribardo D, Jonas L (2009). Mode of expression and functional characterization of FCT-3 pilus region-encoded proteins in Streptococcus pyogenes serotype M49.. Infect Immun.

[23] Godehardt AW, Hammerschmidt S, Frank R, Chhatwal GS (2004). Binding of alpha2-macroglobulin to GRAB (Protein G-related alpha2-macroglobulin-binding protein), an important virulence factor of group A streptococci, is mediated by two charged motifs in the DeltaA region.. Biochem J.

[24] Jonsson H, Lindmark H, Guss B (1995). A protein G-related cell surface protein in Streptococcus zooepidemicus.. Infect Immun.

[25] Lei B, DeLeo FR, Hoe NP, Graham MR, Mackie SM (2001). Evasion of human innate and acquired immunity by a bacterial homolog of CD11b that inhibits opsonophagocytosis.. Nat Med.

[26] Soderberg JJ, von Pawel-Rammingen U (2008). The streptococcal protease IdeS modulates bacterial IgGFc binding and generates 1/2Fc fragments with the ability to prime polymorphonuclear leucocytes.. Mol Immunol.

[27] Akesson P, Moritz L, Truedsson M, Christensson B, von Pawel-Rammingen U (2006). IdeS, a highly specific immunoglobulin G (IgG)-cleaving enzyme from Streptococcus pyogenes, is inhibited by specific IgG antibodies generated during infection.. Infect Immun.

[28] Dinkla K, Sastalla I, Godehardt AW, Janze N, Chhatwal GS (2007). Upregulation of capsule enables Streptococcus pyogenes to evade immune recognition by antigen-specific antibodies directed to the G-related alpha2-macroglobulin-binding protein GRAB located on the bacterial surface.. Microbes Infect.

[29] Mora M, Bensi G, Capo S, Falugi F, Zingaretti C (2005). Group A Streptococcus produce pilus-like structures containing protective antigens and Lancefield T antigens.. Proc Natl Acad Sci U S A.

[30] Okamoto S, Tamura Y, Terao Y, Hamada S, Kawabata S (2005). Systemic immunization with streptococcal immunoglobulin-binding protein Sib 35 induces protective immunity against group: a Streptococcus challenge in mice.. Vaccine.

[31] Timoney JF, Qin A, Muthupalani S, Artiushin S (2007). Vaccine potential of novel surface exposed and secreted proteins of Streptococcus equi.. Vaccine.

[32] Timoney JF, Kumar P (2008). Early pathogenesis of equine Streptococcus equi infection (strangles).. Equine Vet J.

[33] Florindo HF, Pandit S, Goncalves LM, Alpar HO, Almeida AJ (2009). New approach on the development of a mucosal vaccine against strangles: Systemic and mucosal immune responses in a mouse model.. Vaccine.

[34] Florindo HF, Pandit S, Lacerda L, Goncalves LM, Alpar HO (2009). The enhancement of the immune response against S. equi antigens through the intranasal administration of poly-epsilon-caprolactone-based nanoparticles.. Biomaterials.

[35] Morein B, Hu KF, Abusugra I (2004). Current status and potential application of ISCOMs in veterinary medicine.. Adv Drug Deliv Rev.

[36] Hamilton A, Robinson C, Sutcliffe IC, Slater J, Maskell DJ (2006). Mutation of the maturase lipoprotein attenuates the virulence of Streptococcus equi to a greater extent than does loss of general lipoprotein lipidation.. Infect Immun.

[37] Lindmark H, Nilsson M, Guss B (2001). Comparison of the fibronectin-binding protein FNE from Streptococcus equi subspecies equi with FNZ from S. equi subspecies zooepidemicus reveals a major and conserved difference.. Infect Immun.

[38] Lindmark H, Guss B (1999). SFS, a novel fibronectin-binding protein from Streptococcus equi, inhibits the binding between fibronectin and collagen.. Infect Immun.

